# Meeting of the Mississippi Valley Medical Association

**Published:** 1890-11

**Authors:** 


THE meeting of the Mississippi Valley Medical Association, held in Louisville, October 8, 9, and 10, 1890, under the presidency of Dr. Joseph M. Matthews, was a scientific, social, and numerical success. The register contained the names of 167 members on the first day, and many more were added subsequently.

The address, "The Medical Student," by Dr. John A. Wyeth, of New York, was an attractive part of the programme, and the hall where it was delivered proved inadequate to the necessities of the occasion, many reluctantly turning away. An attempt was made to secure another auditorium, but too late to be effectual.

A long list of valuable papers was read and discussed, and the contributions to the literature of medicine will mark an epoch. Many distinguished men were present, among whom we note the names of Drs. Frank Woodbury, Philadelphia; William Porter, St. Louis; Charles A. L. Reed, Rufus B. Hall, J. C. Culbertson,

Edwin Ricketts and G. I. Cullen, of Cincinnati; David Barrow and B. L. Coleman, Lexington; J. C. Sexton, Rushville, Ind.; R. Stansbury Sutton, Pittsburg; John H. Hollister, Chicago; and Dr. Isaac Newton Love, of St. Louis, editor of the *Medical Mirror*, was there *in propria personæ*, to which fact the reflections in the *Mirror* for November will amply testify. The exhibitors of books, instruments and pharmaceutical products were numerous, among whom we note the following prominent houses:

Reed & Carnrick, New York, manufacturers of infant foods and dietetic preparations, represented by Dr. C. C. Fife.

Dietetic Gazette, of New York, represented by Dr. A. H. Still.

Lambert Pharmacal Company, St. Louis, O. H. Dreyser in charge.

H. K. Wampole & Co., Philadelphia, manufacturing druggists, represented by J. E. Langley.

Tarrant & Co., New York, importers Hoff's Malt Extract and manufacturers of Tarrant's Aperient, represented by N. E. Hulbert.

Eli Lilly & Co., pharmaceutical chemists, Indianapolis, represented by Walter H. Evans and J. K. Lilly.

Fairchild Bros. & Foster, New York, manufacturing chemists, represented by Jas. Christie and Jas. Carr.

John Wyeth & Bro., manufacturing chemists, Philadelphia, represented by T. D. Ballard and F. W. McClerkin.

Lea Brothers & Co., medical publishers, Philadelphia, represented by Dr. P. L. Kimball.

D. Appleton & Co., medical publishers, New York, Dr. R. B. Granger, managing editor *New York Medical Journal.*

Wm. R. Warner & Co., manufacturing chemists, Philadelphia, represented by A. D. Roach.

---

The *Dietetic Gazette* announces that one of Reed & Carnrick's extensive factories at Goshen, N. Y., was destroyed by fire on the 10th instant. This factory was devoted wholly to the production of their Soluble Food and Lacto-Preparata, and contained extensive and valuable machinery. They had considerable stock of these foods at their New York office, and consequently there will be no delay in filling orders. The factory will be at once rebuilt three times the size of the one burned, with machinery correspondingly enlarged.

